# Global metabolomics study on the pathogenesis of pediatric medulloblastoma via *UPLC- Q/E-MS/MS*

**DOI:** 10.1371/journal.pone.0287121

**Published:** 2023-06-15

**Authors:** Zhehao Huang, Xianglan Li, Bo Wei, Yin Yu

**Affiliations:** 1 Department of Neurosurgery, China-Japan Union Hospital of Jilin University, Changchun, Jilin, China; 2 Department of Dermatology, China-Japan Union Hospital of Jilin University, Changchun, Jilin, China; Foshan University, CHINA

## Abstract

Medulloblastoma is one of the most frequent malignant brain tumors in infancy and childhood. Early diagnosis and treatment are quite crucial for the prognosis. However, the pathogenesis of medulloblastoma is still not completely clarified. High-resolution mass spectrometry has enabled a comprehensive investigation on the mechanism of disease from the perspective of metabolism. Herein, we compared the difference of metabolic profiles of serum between medulloblastoma (n = 33) and healthy control (HC, n = 16) by using UPLC-Q/E-MS/MS. Principal component analysis and orthogonal projections to latent structures discriminant analysis (OPLS-DA) intuitively revealed the significantly distinct metabolic profiles between medulloblastoma and HC (p < 0.01 for permutation test on OPLS-DA model). Total of 25 significantly changed metabolites were identified. ROC analysis reported that six of them (Phosphatidic acid (8:0/15:0), 3’-Sialyllactose, Isocoproporphyrin, Acetylspermidine, Fructoseglycine and 3-Hydroxydodecanedioate) showed high specificity and precision to be potential diagnosis biomarkers (AUC > 0.98). Functional analysis discovered that there are four pathways notably perturbed for medulloblastoma. These pathways are related with the dysfunction of arachidonic acid metabolism, steroid hormone biosynthesis, and folate-related metabolism. The target intervention on these pathways may reduce the mortality of medulloblastoma.

## Introduction

Medulloblastoma is one of the common malignant brain solid tumors in children [1, 2]. The incidence of this cancer was notably observed in children less than 10 years old [3]. As an embryonal neuroepithelial tumor of the cerebellum, it constitutes around 20% of all intracranial tumors [4]. The early diagnosis of pediatric medulloblastoma could significantly improve the prognosis and avoid severe long-term side effects caused by craniospinal irradiation [5]. The heterogeneity of different subgroups consists of up to 14 molecular subtypes and the tailored therapy could result in improved outcomes [1]. The pathogenesis and heterogeneity of pediatric medulloblastoma is associated with the multiple genetic factors [1, 2] and involves the imbalance of immune microenvironment [6]. However, despite multiple immunotherapies have been tested for medulloblastoma [7], the limited understanding on the mechanism of this rare disease restrict the clinical precision diagnosis and the development of target drug.

Global metabolomics, also named as untargeted metabolomics is a novel cutting-edge approach used to comprehensively detect all small-weight molecules (less than 1 KDa). Compared to other system biology techniques, metabolomics is considered as the best indicator of biological processes [8]. High-resolution mass spectrometry (MS)-based global metabolomics has been extensively used to reveal the complicated diseases [9], including various gliomas [10, 11]. Medulloblastoma has been reported to be highly related with the metabolic changes (e.g. glutamine) [12]. However, there is only one study comparing the metabolic profiles among retinoblastoma, neuroblastoma and medulloblastoma by using NMR platform [13]. The comprehensive detection on the metabolic perturbation in pediatric medulloblastoma is still missing.

Therefore, this study recruited pediatric medulloblastoma patients and healthy children and performed a global metabolomics study via ultra-performance liquid chromatography coupled-Q-Exactive tandem mass spectrometry (UPLC-Q/E-MS/MS) platform. Herein, we tested the hypothesis that there existed significantly distinct metabolic patterns between pediatric medulloblastoma and healthy controls (HC), and the metabolic pathways perturbed in pediatric medulloblastoma contribute to the illustration of the pathogenesis of this rare disease.

## Materials and methods

### Subjects recruitment and ethic assessment

All pediatric patients with medulloblastoma were recruited from the department of neurosurgery, China-Japan Union Hospital of Jilin University between March 1^st^ and December 31^st^, 2019. Patients were diagnosed with computed tomography and magnetic resonance imaging as medulloblastoma for the first time. The diagnosis was finally confirmed with pathological examination of the tumor tissue. Age and gender-matched healthy children were also recruited into the HC group. Patients with other diseases were excluded. All subjects involved in this study provided written informed consent from their supervisors. The demographic and clinical characteristics of all subjects were obtained when samples were collected. This study was evaluated and approved by the China-Japan Union Hospital Committee of Jilin University (No. 2018-NSFC-003).

### Sample collection and processing

Fasting peripheral blood were drawn in the next morning when patients were diagnosed as medulloblastoma. To avoid the variation from circadian rhythms, fasting blood from all HC subjects were also drawn in a morning. Total of 10 mL blood were collected for separation as serum. All serum samples were stored in -80°C for analysis. Once all serum samples were ready, we thawed them in ice and added 1200 *μ*L methanol (Fisher Chemical, UPLC Grade) into the 400 *μ*L serum to precipitate all proteins. The protein-free supernant was lyophilized at -70°C and 10 pa air pressure for 12 hours. Residue was redissolved in 100 *μ*L 80% methanol-water solution. A QC sample was prepared by pooling 15 *μ*L from every serum samples. The QC sample was processed to extract metabolites in the same method as the regular samples. All operations were finished on ice to avoid degradation of metabolites.

### UPLC-Q/E-MS/MS assay

All redissolved samples were injected into a Vanquish UPLC system coupled to a Q Exactive mass spectrometer (Thermo Fisher Scientific, Bremen, Germany). Samples were analyzed using a 17-minute gradients, as described previously [14]. A hydrophobic column (Hypersil GOLD^™^ aQ C18 Polar Endcapped HPLC Column, 100mm × 2.1mm, 1.9μm) were used for reverse phase separation. QC were injected every 8 samples. MS acquisition was performed at MS1 level firstly. Once the acquisition at MS1 level finished, the data preprocessing was performed to get peak table. All significantly changed peaks from univariate analysis were added into the inclusion list for targeted DDA acquisition at MS/MS level.

### Data pre-processing and compound identification

The raw data were converted and centroided with ProteoWizard (v3.0.2) [15] into mzML format. The data pre-processing, including peak picking, alignment, and gap filling were finished with ‘LC-MS Spectra Processing’ module in MetaboAnalyst (v5.0) [16]. All parameters were optimized automatically by the module. Spectra data acquired from MS/MS level were processed with MS-Finder (v3.5) [17]. The MS/MS fragments matching was performed at HMDB [18] and METLIN [19] databases. The threshold of mass error was set as 5 ppm.

### Bioinformatics and statistical analysis

MetaboAnalyst (v5.0) [16] was used to perform bioinformatics and statistical analysis. Raw peak tables were uploaded for multivariate and univariate analysis after normalization with log transformation at ‘Statistical Analysis [one factor]’ module. ROC was performed at ‘Biomarker Analysis’ module. Functional analysis with *mummichog* and GSEA algorithms was performed at ‘Functional Analysis’ module. Pathway database for functional analysis is the mfn database, which is the default option for human functional analysis. Network analysis was performed from the results of functional analysis. Other statistics were finished in R (v4.1) environment.

## Results

### Clinical characteristics

A total of 33 pediatric patients with medulloblastoma and 16 age- and sex-matched healthy children were recruited and included in this study. The clinical demographics of the study cohort are summarized and presented in Table 1. The age and body mass index (BMI) of patients and HC are matched unbiasedly (p > 0.05). The genders within groups are distributed evenly. Only patients of medulloblastoma evaluated at grade M0 and M1 were included [20, 21]. In addition, to balance the influence from the heterogeneity, different subtypes of medulloblastoma were all included in the medulloblastoma group [21].

**Table 1:** Clinical characteristics of all subjects.

	Medulloblastoma	Healthy Control
Subjects Number	33	16
Age/years	7.2±2.4	8.2±3.1
Gender(F/M)	14/19	8/8
Medulloblastoma Grade	M0, n = 23; M1, n = 10	N/A
Subtypes (WNT/SHH/Type3/ Type4)	14/9/4/6	N/A
BMI/ (kg·m^-2^)	22±6.5	23±3.2

Gender includes female (F) and male (M); BMI, body mass index; WNT, Wingless pathway activated subtype; SHH, Sonic Hedgehog pathway activated subtype.

### Multivariate analysis

**Fig 1:**
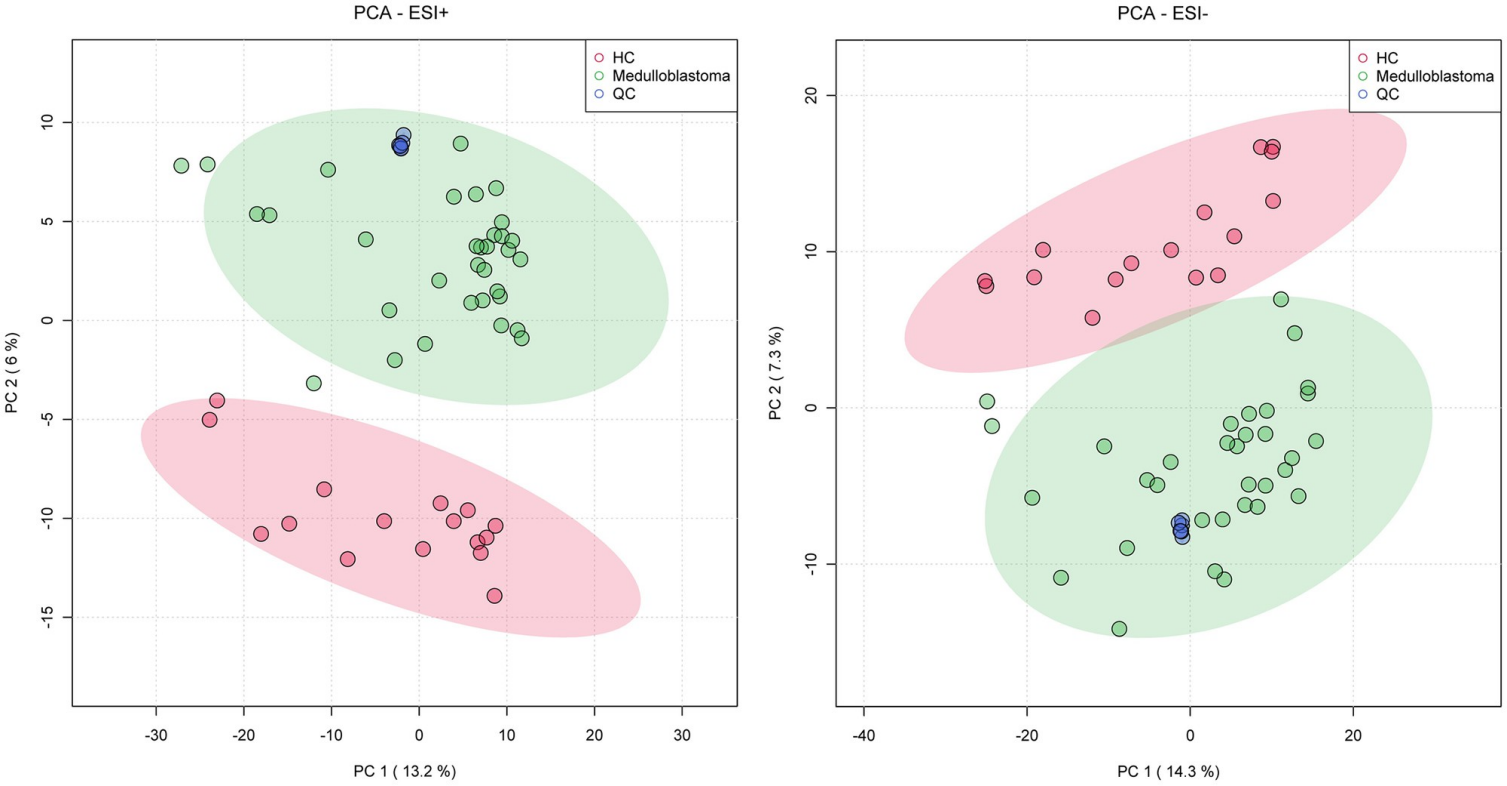
Principal Component Analysis (PCA) score plots of UPLC-MS. 2D PCA score plots of positive ion mode (ESI+) and negative ion mode (ESI-). 95% confidence regions are displayed of every group are displayed. QC, quality control.

A total of 3,319 and 4,450 peaks are detected from the serum of all subjects under positive ion mode (ESI+) and negative mode (ESI-), respectively. The aligned MS peaks were normalized and analyzed with Principal Component Analysis (PCA) and orthogonal projections to latent structures discriminant analysis (OPLS-DA), firstly. PCA, an unsupervised clustering model, displays the general similarity of all samples. As shown in Fig 1, Quality Control (QC) samples are clustered tightly in the score plot for both ESI+ and ESI- modes. This indicates the high stability and precision of the whole chromatography and MS system. The separated clusters of medulloblastoma and HC shows metabolic variation between these two groups. Over 90% samples from patients with pediatric medulloblastoma and all samples from HCs are within the confidence interval (95%). No outliers from medulloblastoma are distributed into HC group, indicating there is that no abnormal sample exists in this study.

**Fig 2:**
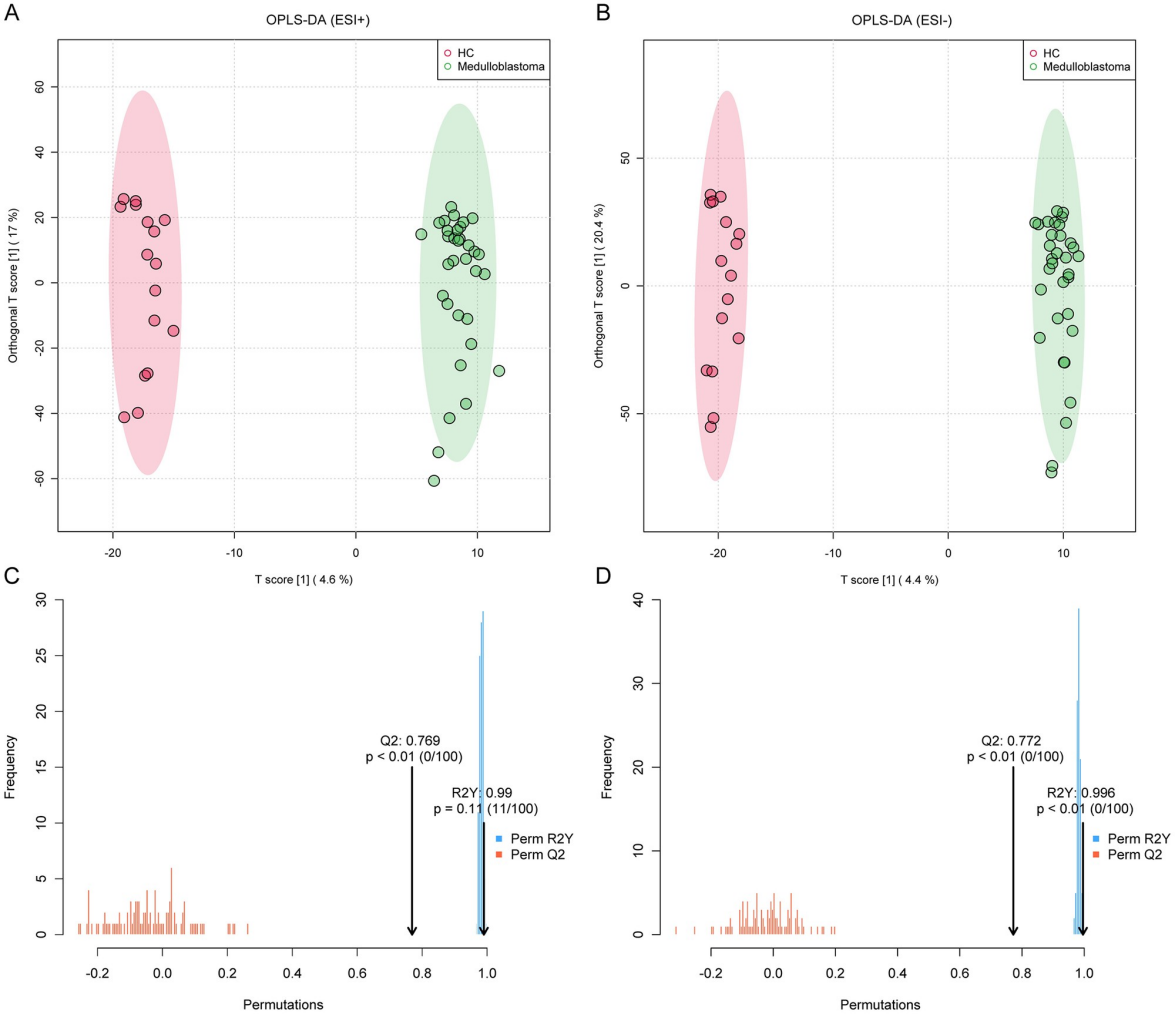
Orthogonal projections to latent structures discriminant analysis (OPLS-DA) score plots and permutation tests results. Quality Control samples are excluded for OPLS-DA. (**A**) Score plot of OPLS-DA (medulloblastoma vs. HC, ESI+). (**B**) Score plot of OPLS-DA (medulloblastoma vs. HC, ESI-). (**C**) Permutation test result of the OPLS-DA of ESI+ (n = 100, R2 = 0.990 and p < 0.01). (**D**) Permutation test result of the OPLS-DA of ESI- (n = 100, R2 = 0.996 and p < 0.01).

As a supervised clustering model, OPLS-DA models are established to discriminate the between medulloblastoma and HC. All features from both ESI+ and ESI- modes are used for OPLS-DA. As shown in Fig 2A and 2B, samples of two groups (medulloblastoma vs. HC) were significantly located on opposite sides and displayed notably explicit separation. This indicates that remarkably different metabolic profiles existed between pediatric medulloblastoma and HC. To ensure the validity and avoid overfitting error, the OPLS-DA models were tested with permutations (n = 100). The permutation results are displayed in Fig 2C and 2D. Q2 of both models are over 0.5 (with p < 0.01), which further confirms the difference of metabolic profiles between medulloblastoma and HC. More details on the distinct metabolic pattern are further clarified in the following section.

### Univariate analysis

**Fig 3:**
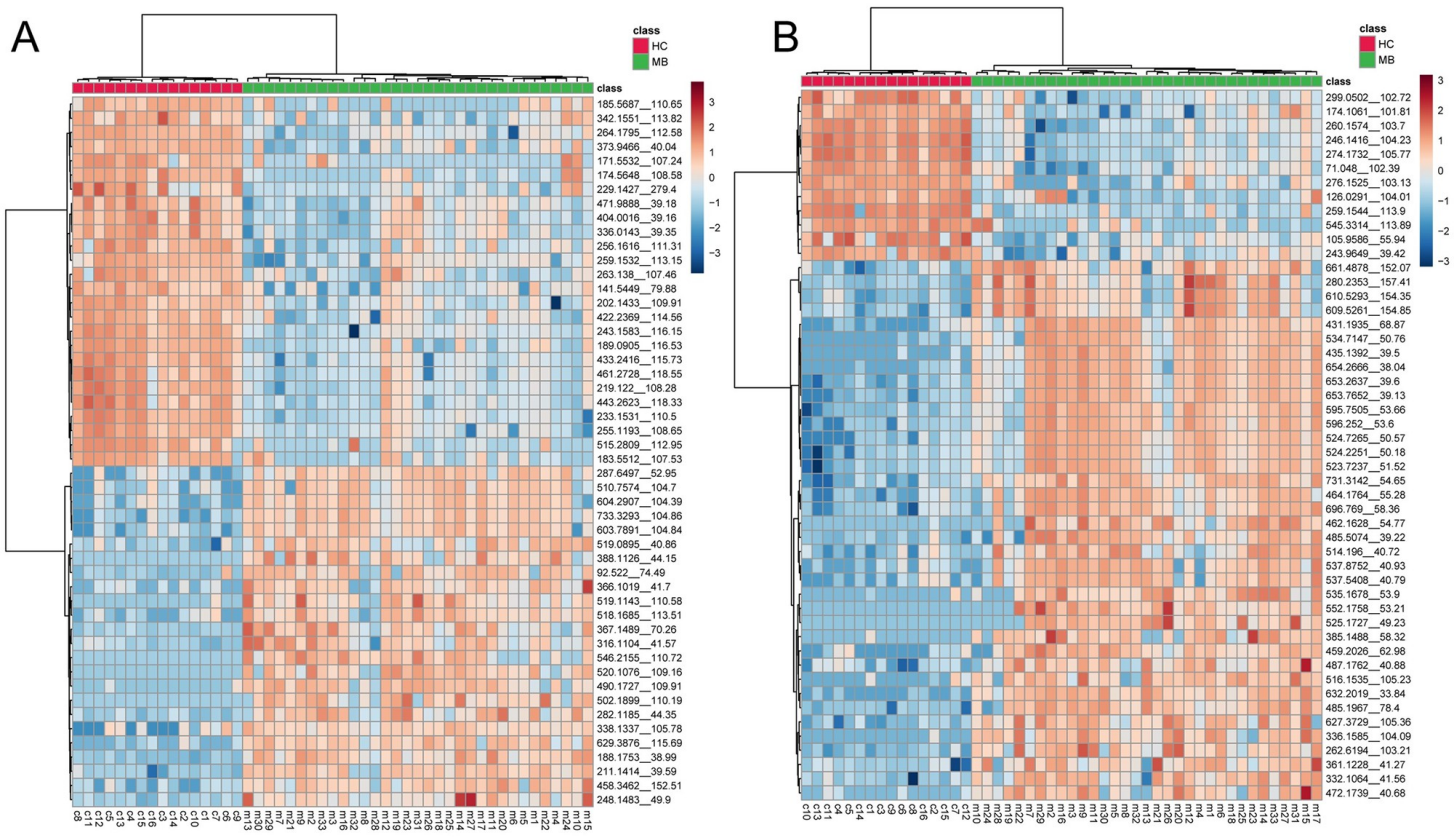
Heatmaps of top 50 significant peaks in both ESI+ (**A**) and ESI- (**B**). Medulloblastoma (MB) and HC can be clustered consistently as the biological groups, indicating an exemption of the abnormal samples. Peaks are labeled as “*m/z__retention time*”. The unit for retention time is second.

The details of the distinct metabolic patterns between medulloblastoma and HC were analyzed with univariate analysis. Top 50 significantly different peaks were extracted and shown as heatmaps (Fig 3). These peaks could clearly cluster the samples as two consistent groups as the disease, which indicate these metabolic peaks mostly illustrate the metabolic difference between medulloblastoma and HC. Targeted data-dependent acquisition (DDA) with tandem MS (MS/MS) was performed by including these peaks as the targets. As a result, total of 25 compounds from the 100 peaks were identified (ppm < 10, Table 2). MS/MS matching patterns of all compounds are provided in S1 Fig in S1 File. All of them are significantly leveled as different intensities between medulloblastoma and HC (*p* < 1×10^−6^), which indicates the potential ability for these compounds to be clinical biomarkers.

**Table 2:** Identified compounds with tandem MS (MS/MS).

NO	m/z	RT/sec	p value	HMDB ID	Adducts	ppm	ESI
1*	188.1753	38.99	1.04E-16	HMDB0002189	M+H	2	+
2	233.1531	110.5	6.87E-14	HMDB0041038	M+H	2	+
3	219.122	108.28	5.79E-13	HMDB0000424	M+H	3	+
4	189.0905	116.53	4.72E-12	HMDB0029495	M+H	3	+
5	243.1583	116.15	1.62E-11	HMDB0030987	M+H	3	+
6*	255.1193	108.65	4.26E-11	HMDB0060278	M+NH4	2	+
7	520.1076	109.16	7.69E-11	HMDB0015059	M+H	2	+
8*	264.1795	122.58	1.15E-10	HMDB0000413	M+NH4	4	+
9	502.1899	110.19	2.81E-09	HMDB0034909	M+NH4	4	+
10	282.1185	44.35	7.23E-09	HMDB0003331	M+H	4	+
11	518.1685	113.51	1.12E-08	HMDB0014464	M+H	4	+
12	202.1433	109.91	1.73E-08	HMDB0036117	M+NH4	2	+
13	422.2369	114.56	5.38E-08	HMDB0012501	M+H-2H2O	0	+
14	229.1427	279.4	7.80E-08	HMDB0000933	M+H	3	+
15	263.138	107.46	1.49E-07	HMDB0014617	M+H	4	+
16*	632.2019	33.84	1.46E-22	HMDB0000825	M-H	4	-
17*	653.7652	39.13	1.04E-14	HMDB0000697	M-H	3	-
18	259.1544	113.9	9.75E-13	HMDB0035028	M-H20-H	1	-
19	485.1967	78.4	1.62E-12	HMDB0000585	M-H	4	-
20*	260.1574	103.7	8.66E-12	HMDB0115486	M-2H	5	-
21	516.1535	105.23	4.23E-10	HMDB0014464	M-H	5	-
22	385.1488	58.32	1.48E-09	HMDB0039570	M-H	4	-
23	459.2026	62.98	2.25E-09	HMDB0010315	M-H20-H	2	-
24	485.5074	39.22	7.35E-09	HMDB0041033	M-H20-H	3	-
25	361.1228	41.27	1.34E-07	HMDB0060116	M+HAC-H	2	-

*, refers to the compounds could be considered as biomarkers for diagnosis.

RT, retention time. HMDB, human metabolite database. ESI, electrospray ionization mode (+, positive; -, negative). P values are tested from t-test.

### Biomarker analysis

**Fig 4:**
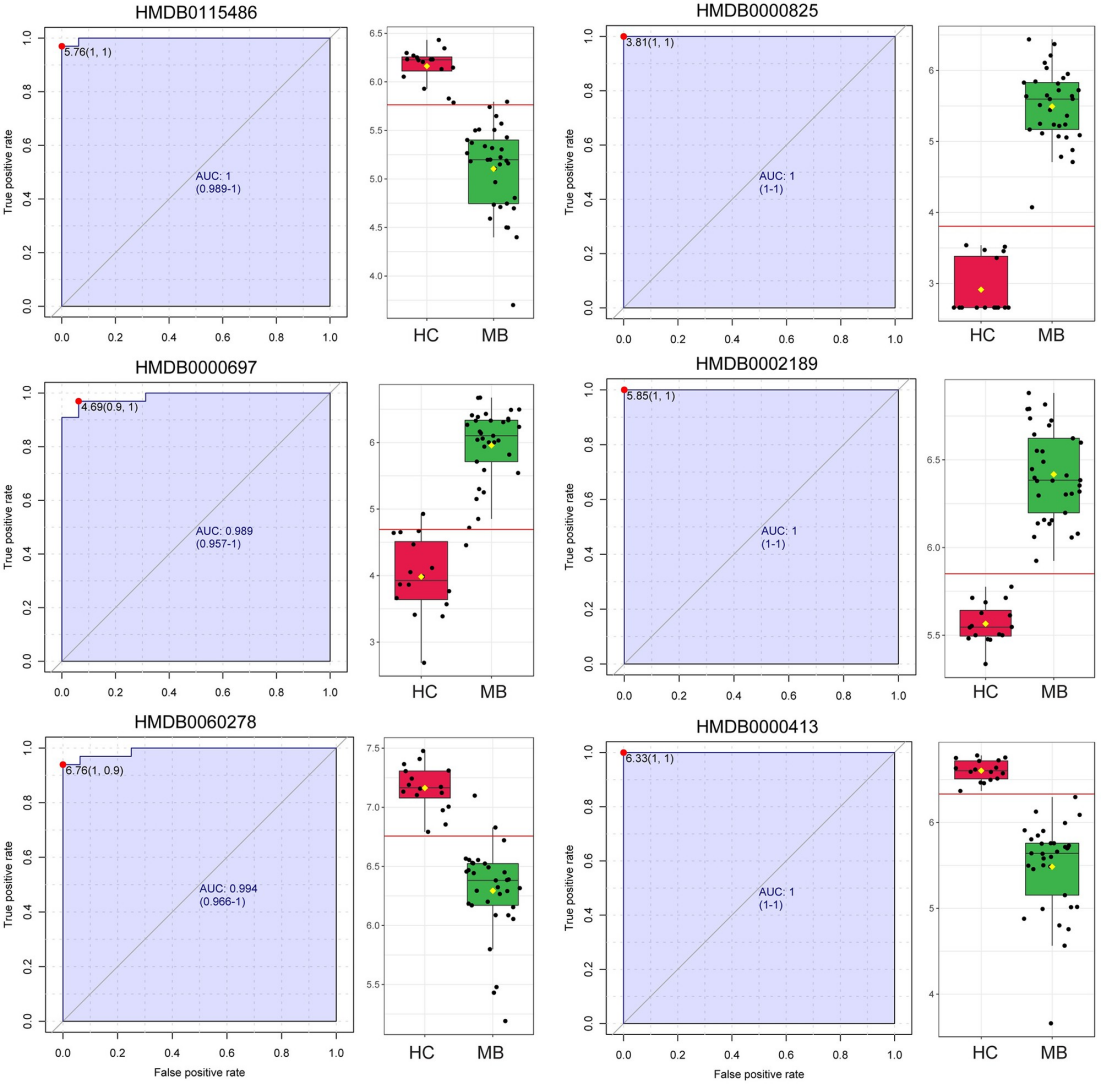
Analysis results of biomarkers with receiver operating characteristic curve (ROC) for medulloblastoma. The ROC curves of all biomarkers are displayed together with boxplots of corresponding peaks’ intensity. HMDB0115486 is Phosphatidic acid (8:0/15:0). HMDB0000825 is 3’-Sialyllactose. HMDB0000697 is Isocoproporphyrin. HMDB0002189 is Acetylspermidine. HMDB0060278 is Fructoseglycine. HMDB0000413 is 3-Hydroxydodecanedioate. AUC, area under curve. HC, healthy control. MB, medulloblastoma.

All peaks from both ESI modes were used to evaluate to be biomarkers with receiver operating characteristic curve (ROC). We set the threshold of biomarkers as AUC > 0.98. As a result, total of 11 peaks in ESI+ modes and 16 peaks in ESI- modes are reported as potential biomarkers. Targeted DDA MS/MS identified six compounds (Table 2 and Fig 4). There are three metabolites (3’-Sialyllactose, Isocoproporphyrin and Acetylspermidine) remarkably increased in medulloblastoma. The other three metabolites, including Phosphatidic acid, Fructoseglycine and 3-Hydroxydodecanedioate, are tremendously decreased in medulloblastoma compared to HC. Both sensitivity and specificity of all six biomarkers are over 90%. The fold changes of all biomarkers are summarized in S1 Table in S1 File.

### Functional analysis

**Fig 5:**
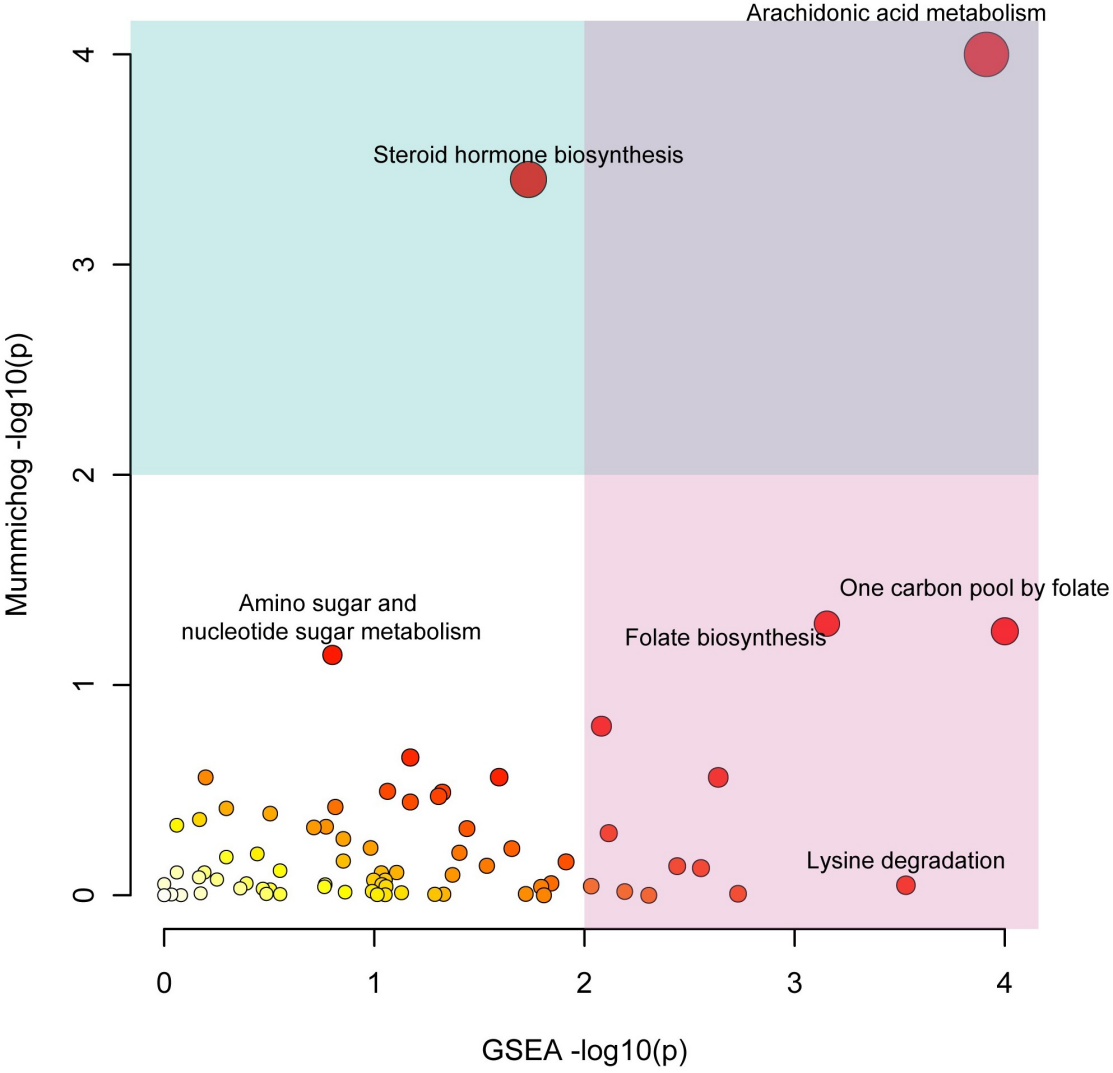
Function analysis result. Functional analysis was performed with *mummichog* and Gene Enrichment Analysis (GESA) algorithm, simultaneously. The p values are log-transformed and scaled for x-axis and y-axis, respectively. There are total of six significantly perturbed pathways discovered.

Functional analysis was performed by using both *mummichog* and gene enrichment analysis (GSEA) algorithms simultaneously [16, 22]. The perturbed pathways are reported by merging p values from both algorithms. As shown in Fig 5, there are six significantly perturbed pathways found in medulloblastoma. Four of them (arachidonic acid metabolism, steroid hormone biosynthesis, one carbon pool by folate, folate biosynthesis) are discovered as highly significant (merged p < 0.05, Table 3). The other two pathways (amino sugar and nucleotide sugar metabolism, lysine degradation) are relatively significant (p < 0.05 for *mummichog* or GSEA).

**Table 3:** Functional perturbation results for medulloblastoma compared to healthy control.

Pathways	Total	Sig_Hits	*Mummichog*_p	GSEA_p	Combined_p
Arachidonic acid metabolism*	27	11	2.00E-05	0.02222	1.00E-05
Steroid hormone biosynthesis*	149	30	1.00E-04	0.1852	0.00021
One carbon pool by folate*	13	4	0.03347	0.02041	0.00566
Folate biosynthesis*	45	9	0.03036	0.04651	0.01068
alpha-Linolenic acid metabolism	6	2	0.1135	0.1321	0.07795
Porphyrin metabolism	41	6	0.2194	0.07692	0.08577
Amino sugar and nucleotide sugar metabolism^#^	71	12	0.04539	0.459	0.1015
Lysine degradation^#^	99	7	0.8786	0.03226	0.1293
Drug metabolism	130	16	0.2187	0.2121	0.1888

GSEA, Gene enrichment analysis.

*, highly significant (merged p < 0.05).

^#^, relatively significant (p < 0.05 reported by *mummichog* or GSEA).

Sig_Hits, the pathway hits from significantly changed compounds. *Mummichog*_p, p value from *mummichog* algorithm. GSEA_p, p value from GSEA algorithm. Combined_p, merged p value of *mummichog* and GSEA algorithms.

### Network analysis

**Fig 6:**
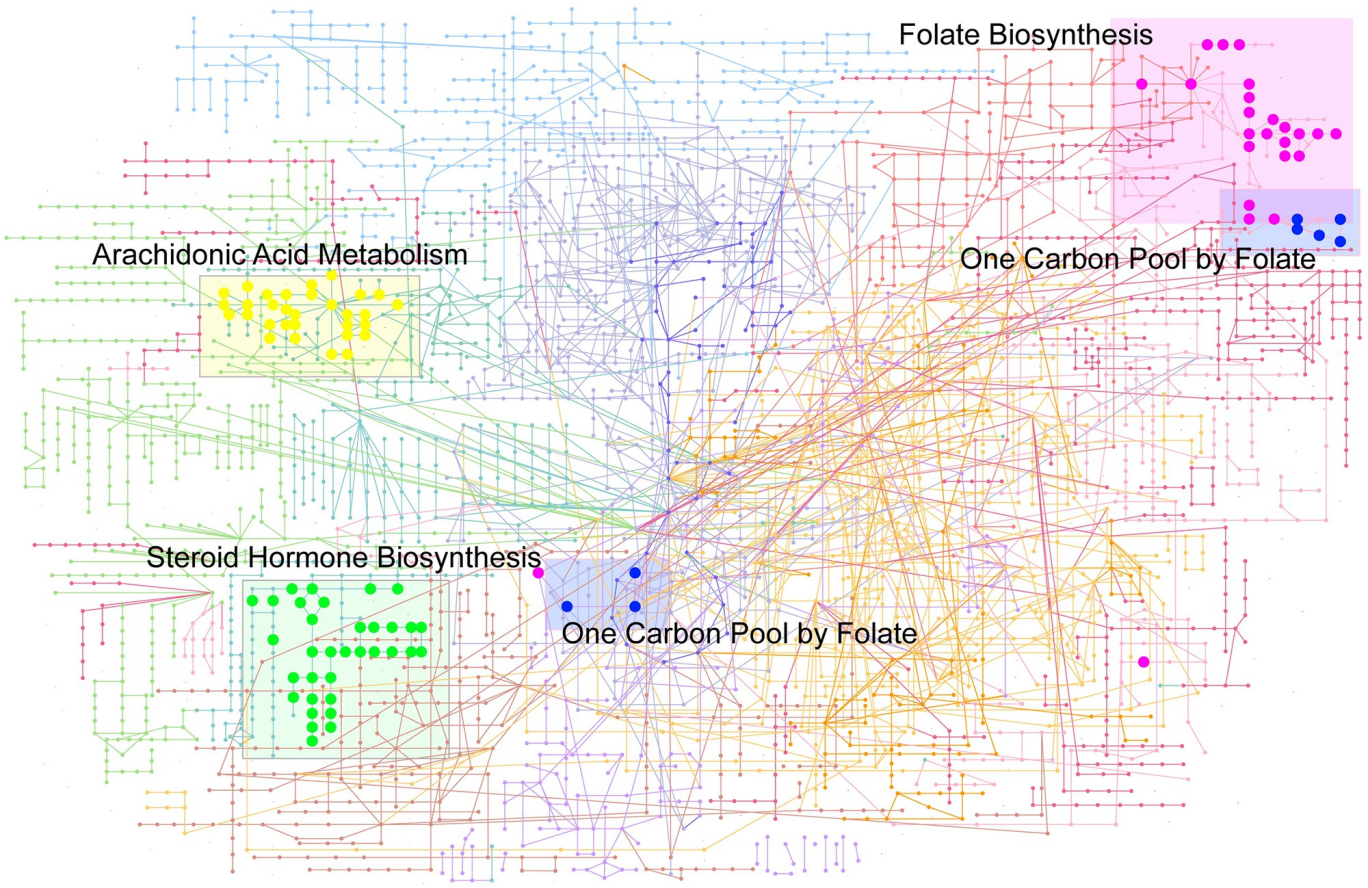
Global network overview of all perturbed metabolic pathways and their corresponding metabolites. Only the highly significantly perturbed four pathways are shown. The mapped network shows the relationships among all remarkably changed functional pathways.

In order to gain a high-level overview of the metabolic activities in medulloblastoma, all highly perturbed functional pathways were mapped into the KEGG global network (Fig 6) [23]. The matched network visually reflects the influence on the whole metabolic network. Besides, displaying functional pathways at network level could intuitively show the coordination among different perturbed pathways [24]. As shown in Fig 6, arachidonic acid metabolism and steroid hormone biosynthesis are changed independently from other pathways, while one carbon pool by folate are obviously coupled with folate biosynthesis pathway.

## Discussion

Medulloblastoma has been reported as the second most common pediatric tumor occurring in central nervous system [25]. In this study, we performed a comprehensive metabolomics study to investigate the metabolic difference between medulloblastoma and HC. The metabolic profile of medulloblastoma is significantly different from HC. Total of 25 significantly changed metabolites were identified, while six of them showed high specificity and precision as diagnosis biomarkers. Besides, four functional pathways were discovered as significantly perturbed.

Implementation of metabolomics techniques in elucidating the pathogenesis of glioma is emerging [26] with the development of high-resolution mass spectrometry, but it is rarely utilized to clarify the pathogenesis of medulloblastoma. To our knowledge, there are only a few studies focusing on the metabolic profiles of medulloblastoma [13, 27–29]. However, most of them were performed with NMR platform. The coverage of the whole metabolome is too low to comprehensively display the metabolic difference between the corresponding comparisons (e.g., with other tumors or controls). Ji HL *et al*. analyzed the metabolic profiles of cerebrospinal fluid from the patients with multiple brain tumors (including medulloblastoma) but did not report differentiated metabolic patterns [26]. UPLC-MS/MS based metabolomics provides a higher coverage on the metabolome. It has become the workhorse for untargeted metabolomics studies. Besides, compared to NMR, UPLC-MS/MS shows higher metabolome coverage and sensitivity [30]. Therefore, we applied UPLC-MS/MS based untargeted metabolomics in the present study to reveal more metabolic changes. Generally, it is the first time to comprehensively clarify the metabolic difference in serum between medulloblastoma and healthy control in the present study.

In clinical practice, most children with medulloblastoma are treated with maximal surgical resection, chemotherapy, and radiation therapy [31]. However, the mechanism of medulloblastoma is still unknown. More importantly, the deficiency of reliable clinical diagnosis biomarker makes the diagnosis specifically relying on the imaging examination, like magnetic resonance imaging and computerized tomography [32, 33]. Highly accurate biomarkers could be helpful for early pre-diagnosis in the regular peripheral blood-based biochemical screening. However, blood-based diagnosis has to depend on the external mediate, like synthetic alloys at current stage [34]. Herein, we identified six compounds, which showed high performance to work as diagnosis biomarkers in blood. One of them is a non-polar lipid (phosphatidic acid) and other five belong to polar metabolite..

Total of four functional pathways reported as significantly perturbed with high confidence in this study. Arachidonic acid metabolism is a biological process metabolizing arachidonic acid metabolism via cyclooxygenase and lipoxygenase into pro-inflammatory prostanoids and leukotrienes [35]. This metabolic pathway has been extensively reported to enroll various diseases, including cardiovascular diseases [36], inflammatory processes [37] and tumors [38]. Arachidonic acid metabolism is directly associated with the pathogenesis of medulloblastoma [39] and the occurrence of drug resistance [40]. Increased leukotriene synthesis could significantly contribute to the progression of medulloblastoma [41]. Prostaglandin E_2_ could also be used as the therapeutic target for medulloblastoma [42]. Besides, the change of arachidonic metabolism associated metabolites in micro-environment of cancers may be observed to contribute to the recurrent medulloblastoma [43]. This study further confirms the role of arachidonic acid metabolism pathway in the pathogenesis of medulloblastoma. This result is consistent to the reports from urine samples [29].

As a key component of endocrine [44], steroid hormone biosynthesis involves tremendous biological activities, such as the development of organs [45]. Steroid hormone biosynthesis is also associated with the prognosis of multiple cancers, like prostate cancer [46]. Multiple steroid hormone related genes [47] and receptors [48] have been observed as differentially expressed in medulloblastoma. Similar perturbation of steroid hormone biosynthesis pathway has also been observed in the another recent metabolomics study on medulloblastoma from urine samples [29]. A chemical component in the steroid hormone biosynthesis pathway, cortisol has been demonstrated to inhibit the DNA repair [49], and may contribute to the pathogenesis of medulloblastoma [50]. Targeted intervention on the biosynthesis may be a potential therapeutic strategy for medulloblastoma.

Folate is an essential nutrient for animals. But human is lacking the ability to do biochemical synthesis de novo. Folate participates in the growth and development of human [51]. The deficiency of folate may cause the defects of neural system [52]. Folates constitute a critical one-carbon pool to regulate the biosynthesis of other bio-active metabolites, and thereby intervening cellular proliferation and tissue homeostasis [51, 53]. Previous studies have reported the influence of dietary folate [54] and expression level of folate receptor 1 [55] on the formation of medulloblastoma. Consistent with previous study, this study further demonstrates the metabolic perturbation of folate metabolism in medulloblastoma. Folate metabolism pathways could be another potential therapeutic target.

Besides, there are two marginal significant pathways reported as changed in medulloblastoma. The relationship between medulloblastoma and amino/nucleotide sugar metabolism has not been reported before. Similarly, how lysine degradation affects the pathogenesis of medulloblastoma is still unclear. The functional perturbation of these two pathways needs further validation with a larger cohort. Besides, it is noted that the perturbed pathways reported in the present study is similar to the ones reported by another urine-based metabolomics [29], indicating that a systematic metabolic change occurs in medulloblastoma. Pathway analysis is based on mfn database, which contains regular biological processes but may be limited in the coverage of metabolic functions. This untargeted metabolomics reveals a general metabolic perturbation in medulloblastoma. All identified function pathways need further investigation on the changes of each compound in the pathway in a future targeted metabolomics study. Despite medulloblastoma is the second most solid tumor of central nerve system for children, the incidence of this disease is still very rare (overall average crude incidence rate was 0.12 per 100,000 [56]). The major limitation of this study is the sample size, which may need multicenter clinical research worldwide in the future. In addition, this study only includes the pediatric patients with medulloblastoma at M0 and M1 grade. Other more severe grades (M2-M4) should also be included for future study.

## Conclusion

In this study, a comprehensive global metabolomics analysis was implemented to reveal the metabolic profiles in pediatric medulloblastoma. Our findings indicate that there exists significant metabolic perturbation for children with medulloblastoma. Phosphatidic acid (8:0/15:0), 3’-Sialyllactose, Isocoproporphyrin, Acetylspermidine, Fructoseglycine and 3-Hydroxydodecanedioate could be used as the biomarkers for early diagnosis. Our results suggest that extensive abnormality of arachidonic acid metabolism, dysregulation of steroid hormone biosynthesis, and changed folate-related metabolism are the metabolic characteristics underlying the progression of medulloblastoma. The target intervention on these pathways may contribute to the improvement of survival rate.

## Supporting information

S1 File Contains all the supporting tables and figures. (DOCX)
