# Supplementary material for: Global metabolomics study on the pathogenesis of pediatric medulloblastoma via UPLC- Q/E-MS/MS
Source: PLoS One. 2023 Jun 15;18(6):e0287121. doi: 10.1371/journal.pone.0287121 (PMC10270352; doi:10.1371/journal.pone.0287121)
Supplement: S1 File — (DOCX) [file pone.0287121.s001.docx]

Supplementary materials

**Global Metabolomics Study on the Pathogenesis of Pediatric Medulloblastoma via *UPLC- Q/E-MS/MS***

Zhehao Huang ^1, †^, Xianglan Li ^2, †^, Bo Wei ^1^ and Yin Yu ^1,^*

1. Department of Neurosurgery, China-Japan Union Hospital of Jilin University, Changchun 130033, Jilin, China;

2.Department of Dermatology, China-Japan Union Hospital of Jilin University, Changchun 130033, Jilin, China;

* Correspondence: yuyin@jlu.edu.cn; † These authors contributed equally to this work.


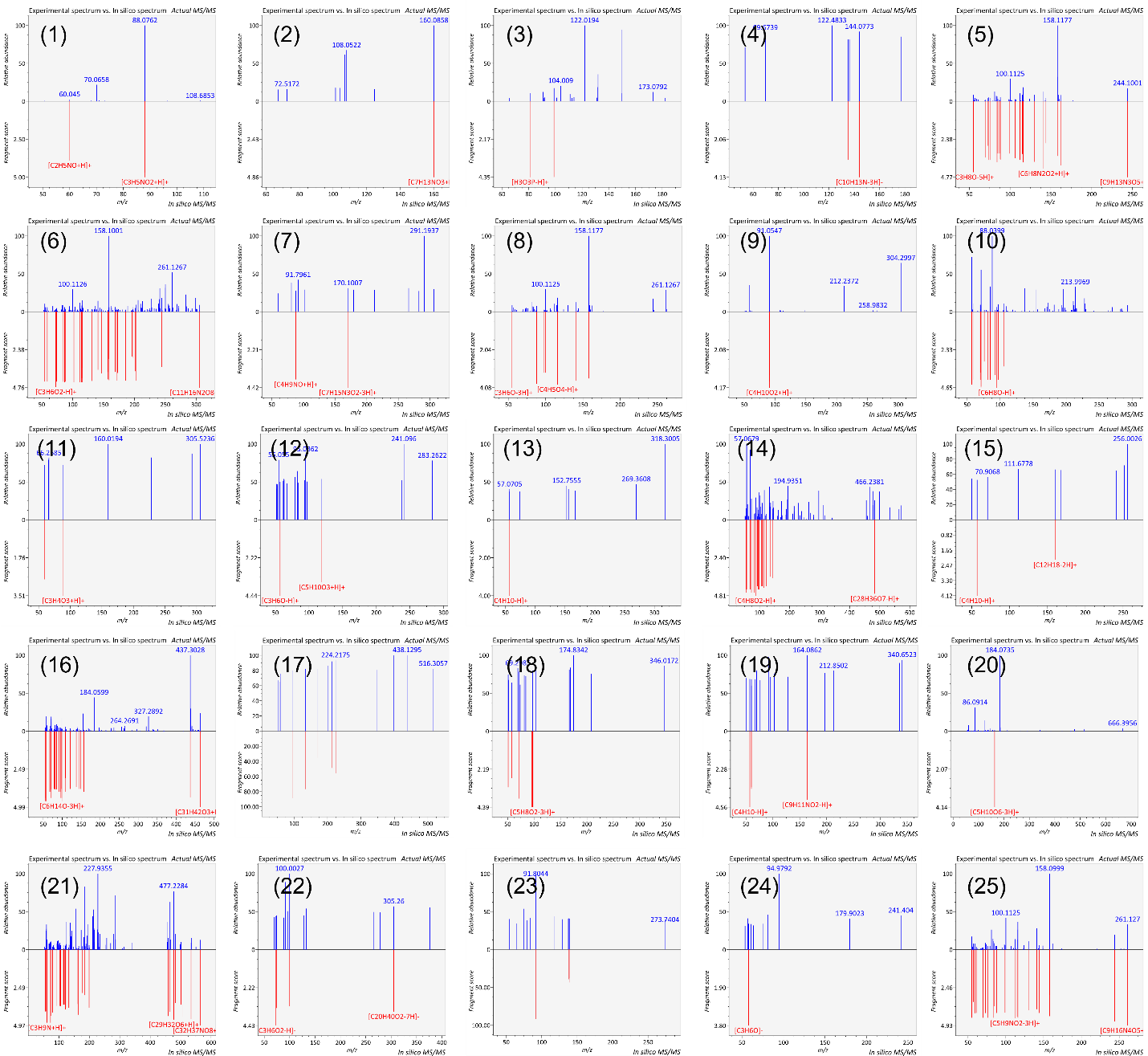


**Figure S1.** MS/MS matching pattern of all identified compounds. These images are exported from MS-FINDER (v.3.5.2).

**Table S1.** Fold changes of all biomarkers

| **Biomarkers** | **Fold change (HC/MB)** |
| --- | --- |
| HMDB0115486 | 8.709 |
| HMDB0000825 | 0.0022 |
| HMDB0000697 | 0.0060 |
| HMDB0002189 | 0.2138 |
| HMDB0060278 | 6.165 |
| HMDB0000413 | 9.772 |
